# Editorial: Metaverse going beyond adoption: the next frontier for global healthcare

**DOI:** 10.3389/fpubh.2023.1194285

**Published:** 2023-05-02

**Authors:** Umer Zaman

**Affiliations:** Endicott College of International Studies, Woosong University, Daejeon, Republic of Korea

**Keywords:** global healthcare, metaverse, mental health, virtual reality, telepresence, virtual hospitals, artificial intelligence, augmented reality

The technological convergence of major trends and alternate realities (i.e., virtual reality, mixed reality, augmented reality and artificial intelligence) through Metaverse is demonstrating enormous potential to revolutionize global healthcare (1, 2, 4). The Metaverse is a virtual world that is becoming increasingly popular with many industries, including healthcare (1, 4), that are exploring its extended applications and benefits (e.g., fully immersive and hyper-realistic virtual interface between healthcare professionals and patients) (1, 3, 6). Metaverse creates amazing possibilities for healthcare professionals (e.g., remote consultations and telemedicine) to create a transformational impact on patient's health, regardless of their location, including healthcare accessibly in underserved communities (3, 4). Importantly, Metaverse accelerates healthcare education on a global scale (1, 3), as healthcare professionals can create virtual training programs that allow them to simulate medical procedures and techniques (2, 3). This provides a safe and controlled environment for healthcare experts to practice their skills, which can significantly improve patient outcomes through remote monitoring (3, 4). Metaverse-based platforms can improve healthcare by providing new channels (e.g., telepresence) for healthcare professionals to virtually connect with their patients (using customized avatars) and deliver 24/7 healthcare assistance (1, 2). In many parts of the world, access to healthcare is limited, with patients having to travel long distances to receive medical care. Hence, Metaverse can create a more personalized and engaging experience for patients by overcoming all forms of geographical barriers (1, 3, 4).

Metaverse represents a virtual world created through the convergence of both digital and physical worlds (2, 3). However, there are several challenges that need to be addressed, to ensure that the Metaverse is secure and safe for healthcare applications (2, 4), and it is accessible to patients across the globe (3). While the Metaverse has the potential to improve access to healthcare in underserved communities, there is an underlying risk that it could widen the digital divide, with some patients being unable to access healthcare in the Metaverse. If these challenges can be addressed, the Metaverse has the potential to transform global healthcare for the better (2, 3). This primer aims to highlight the radical transformation of global healthcare with the emergence of Metaverse technologies.

The scope of this Research Topic entitled “*Metaverse Going Beyond Adoption: Next Frontier for Global Healthcare*” in the Frontiers in Public Health, was to stimulate and encourage research toward the emergence of the Metaverse and its potential implications for global healthcare (1, 2). This Research Topic draws scholarly attention toward the review of academic literature, especially in the wake of the COVID-19 pandemic that focuses on ground-breaking opportunities and challenges for global healthcare with the emergence of the Metaverse (1, 4). The Research Topic was open to any work (including qualitative, quantitative, mixed-methods, empirical assessments, case studies, methodological or review papers, underpinning/grounded theories, policy directives and analysis) related to the adoption and applications of Metaverse (including artificial intelligence, digital twinning, blockchain technology, augmented reality, cybersecurity, virtual reality, telepresence, virtual hospitals and digital technologies convergence) in the global healthcare. Each paper published within this Research Topic made original contributions by examining the diverse contexts of global healthcare within Metaverse. Zang et al. explored mental health and virtual reality blended with project-based learning (PBL) experience of high school students (in China) to increase their academic performance. Zhang and He examined the health consciousness in extreme sports based on college students' innovation resistance and exposure to advertising in augmented reality. Huang et al. developed a mediating model of mental health to examine the endurance performance of young Chinese athletes through performance anxiety and virtual reality experience in Metaverse. Sohail et al. employed partial least squares structural equation modeling (5) as well as artificial neural networks (ANN) to test and validate the predictors of public awareness about COVID-19 (including health risk perception, social distance, mask usage, protective measures and personal hygiene). Lastly, Hu and Sheng utilized the system theory to explore blockchain technologies in an extended cross-chain collaboration model linked with various implications for the medical health information and chained interactions (including diagnosis, rehabilitation, treatment, prevention, and detection) for older adults.

## Author contributions

The author confirms being the sole contributor of this work and has approved it for publication.
